# Periscope.—The Blister-Treatment of Rheumatism

**Published:** 1873-11

**Authors:** 


### PERISCOPE.—THE BLISTER-TREATMENT OF RHEUMATISM.

Dr. T. B. Peacock, of St. Thomas' Hospital, says in the *British Medical Journal:—*

I have now been in the regular use of the blister-treatment of rheumatism since 1865. When I first employed it, it was only tentatively, one, two, or three blisters being applied at the same time or in succession, and in conjunction with other remedial means, and the general impression which I formed was not very favorable. Subsequently, I was induced to apply the blisters much more freely, three or four, or even six, at a time, and in rapid succession a still larger number, and I have been led to form a high opinion of their usefulness when thus used, and to confirm what has been said in favor of the treatment by Dr. Davies. The blisters are generally two or three inches wide, and sufficiently long to encircle the limb. They are placed above the chief joints that are affected, and are usually put on in the after part of the day; in the morning, or when they have risen sufficiently, the serum is let out and the surfaces covered with warm linseed-meal poultices, and these are continued for several days. The treatment has been objected to as unnecessarily severe and attended with much suffering to the patient, but this is not correct. I scarely remember an instance in which the patient, though specially questioned on the subject, has found fault with the treatment; and I have often heard them say that the pain caused by the blister is not to be compared with that of the rheumatism. Nor have I seen any serious inconvenience of any other kind caused by the blisters. Sometimes, however, there is a temporary increase of suffering when the blisters begin to draw, and the temperature rises, and the patients are restless at night; but generally there is a very marked amendment in the morning, both the swelling, tenderness, and pain being reduced, and the constitutional disturbance relieved. In some cases, however, the local symptoms may not be immediately benefitted to any marked degree, and the blisters must be repeated, being applied above to the seat of the first vesication; or, after a few days' cessation, the same joint may be again affected, and in this case too the blistering must be repeated. The occurrence of second attacks in the joints first affected is not, however, by any means confined to cases treated by blisters, but equally occurs when constitutional means have been had recourse to.

Generally with the local means, constitutional remedies, especially the bicarbonate and nitrate or tartrate of potash, are given more or less freely, according to the severity of the symptoms. The cases in which I have tried the blister-treatment are the following:

First, when several joints are coincidentally and severely affected, the sufferings of the patient are great, the constitutional disturbance severe, and the temperature high; in cases of this kind, three, four, or even six or more blisters are applied immediately the patient is seen, and as many more may be put on in the course of a few days, in rapid succession, as other joints are involved, or when those first blistered are not materially relieved or again become affected. From this treatment I have seen the most satisfactory results, both the local and general symptoms being greatly relieved by the free blistering, and the duration of the disease being curtailed. It is evident also that, if the active stage of the disease be shortened, as this is the period during which the internal complications are most apt to occur, the frequency of the complications will be lessened. It is in cases of this kind that the blister treatment is most efficacious, the benefit obtained being apparently directly proportionate to the number of the joints coincidentally affected, to the severity of the local symptoms, and to the freedom with which the blisters are applied to the whole of the parts involved, so that an immediate and decided impression is produced upon the disease. In cases where only two or three joints are affected, though these may be all blistered, the relief obtained to the constitutional disturbance is less decided, and where the pains are rather diffused over all parts of the body than limited to certain joints, the remedy cannot be satisfactorily employed. I have mentioned that the occurrence of internal complications may be prevented by the early and free employment blistering; but in some cases we have proof of much more decided improvement being produced, for I have seen cases in which there were very threatening symptoms of cardiac disturbance, such as are ordinarily followed by serious disease of the pericardium or valves, entirely relieved by the free blistering of the inflamed joints, and the cardiac symptoms apparently arrested.

In cases of this kind, the free discharge from the vesicated surfaces operates apparently as an outlet to the *materies morbi*, and so causes the disease to exhaust itself on the external and less important parts of the body. So satisfied have I been with the effects of the blister-treatment in cases of intense rheumatic fever, that I have gradually reduced the use of the internal remedies, giving much smaller doses of the bicarbonate or nitrate of potash, or only employing coincidentally some slight diaphorectic, as the tartrate of potash.

Secondly, I have known very satisfactory results from the blister-treatment in cases in which the symptoms, both constitutional and local, were less severe, but where the patient's strength was greatly reduced either from previous attacks of rheumatism or other cause, or when the heart was already seriously diseased. In cases of this kind the use of remedies which exercise any depresing influences is to be avoided if possible. I have, therefore sometimes relied on the blister-treatment alone, or in combination with tonics, quinine and iron, and with very good results. The blisters, even though freely applied, do not depress the strength so much as the use of alkalies or other constitutional remedies. When the heart is diseased from a previous rheumatic attack, and when, as is generally the case, the patient is very anæmic, the use of depressing remedies is especially objectionable. In such cases, also, the attack should be arrested as quickly as possible, lest the heart should become again involved; and I know no means so likely to accomplish this as the free blistering of all the affected joints.

Thirdly, another class of cases, in which the rheumatic affection rather involves the smaller joints, what is often called rheumatic gout, and in which the constitutional disturbance is of a more subacute character, is also very often benefitted by the use of blisters, though less decidedly than the two other forms of disease. In cases of this kind the blisters need not, however, be employed so freely as in the former cases; I also generally combine them with the internal administration of small doses of iodide of potassium, bicarbonate of potash and colchicum, and often with bark or quinine. As we all know, cases of this kind are very apt to be tedious, whatever be the plans of treatment which we adopt; but I believe that the combination of local and general remedies which I have named is generally the most efficacious means of relief.

Lastly, there are cases in which the disease rather assumes the neuralgic than the ordinary rheumatic form, where the pains follow the course of certain nerves, and are often very persistent, in which the application of blisters is very beneficial. The treatment is a very old one, but it is one which has perhaps of late years received less attention than it deserves.—*Med. and Surg. Reporter.*

